# Maternal death review and surveillance: The case of Central Hospital, Benin City, Nigeria

**DOI:** 10.1371/journal.pone.0226075

**Published:** 2019-12-19

**Authors:** Josephine Aikpitanyi, Victor Ohenhen, Philip Ugbodaga, Best Ojemhen, Blessing I. Omo-Omorodion, Lorretta FC Ntoimo, Wilson Imongan, Joseph A. Balogun, Friday E. Okonofua

**Affiliations:** 1 The Women’s Health and Action Research Centre (WHARC), Benin City, Nigeria; 2 The Centre of Excellence in Reproductive Health Innovation (CERHI), University of Benin, Benin City, Nigeria; 3 The Central Hospital, Benin City, Nigeria; 4 The Federal University, Oye-Ekiti, Nigeria; 5 The University of Medical Sciences, Ondo City, Ondo State, Nigeria; 6 Chicago State University, Chicago, United States of America; African Population and Health Research Center, KENYA

## Abstract

**Background:**

Despite the adoption of Maternal and Perinatal Death Surveillance and Response (MPDSR) by Nigeria’s Federal Ministry of Health to track and rectify the causes of maternal mortality, very limited documentation exists on experiences with the method and its outcomes at institutional and policy levels.

**Objective:**

The objective of this study was to identify through the MPDSR process, the medical causes and contributory factors of maternal mortality, and to elucidate the policy response that took place after the dissemination of the results.

**Methods:**

The study was conducted at the Central Hospital, Benin between October 1, 2017, and May 31, 2019. We first developed a strategic plan with the objective to reduce maternal mortality by 50% in the hospital in two years. An MPDSR committee was established and the members and all staff of the Maternity Department of the hospital were trained to use the nationally approved protocol. All consecutive cases of maternal deaths in the hospital were then reviewed using the MPDSR protocol. The results were submitted to the hospital Management and its supporting agencies for administrative action to correct the identified deficiencies.

**Results:**

There were 18 maternal deaths in the hospital during the period, and 4,557 deliveries giving a maternal mortality ratio (MMR) of 395/100,000 deliveries. This amounted to a seven-fold reduction in MMR in the hospital at the onset of the project. The main medical causes identified were obstetric hemorrhage (n = 10), pulmonary embolism (n = 2), ruptured uterus (n = 2), eclampsia (n = 1), anemic heart failure (n = 1) and post-partum sepsis (n = 2). Several facility-based and patient contributory factors were identified such as lack of blood in the hospital and late reporting with severe obstetric complication among others. Response to the recommendations from the committee include increased commitment of hospital managers to immediately rectify the attributable causes of deaths, the establishment of a couples health education program, mobilization and sensitization of staff to handle pregnant women with great sensitivity, promptness and care, the refurbishing of an intensive care unit, and the increased availability of blood for transfusion through the intensification of blood donation drive in the hospital.

**Conclusion:**

We conclude that the results of MPDSR, when acted upon by hospital managers and policymakers can lead to an improvement in quality of care and a consequent decline in maternal mortality ratio in referral hospitals.

## Introduction

Maternal death is one of the most critical public health and developmental challenges globally, especially in the least developed regions where maternal mortality remains very high [1]. The lifetime risk of a woman dying from a pregnancy-related cause in a low-income country is 25 times higher than in a high-income country [2]. In 2004, the World Health Organisation (WHO), in a landmark publication titled “Beyond the Numbers”, made a recommendation that all the countries that had not put a medical auditing system in place for the reduction of maternal deaths, to do so without delay [1]. The WHO describes five main approaches to ascertain the causes and contributory factors for maternal deaths and ill health [1, 2, 3]. One of these is the facility-based maternal death review which entails the auditing of all maternal deaths that occur in health facilities. The facility-based maternal death review is an in-depth investigation of the causes and contributory factors in maternal deaths that occur in health facilities [3]. A maternal death review is based on the surveillance cycle which consists of the identification of maternal deaths, data collection, analysis of findings, recommendations, action, evaluation, review, and feedback [3].

In Nigeria, maternal death was estimated to be 58,000 in 2015 accounting for 14% of the global maternal deaths [4]. The high maternal mortality in Nigeria remains despite several measures such as maternal and perinatal death review put in place by the government [5]. A large proportion of maternal deaths in Nigeria as in many low-resource countries result from poorly managed deliveries, in particular when obstetrical complications occur [6, 7, 8]. Treatments for these complications are well established and appropriate emergency obstetric care can prevent most maternal deaths [9]. Maternal death has devastating effects on the family and long-term consequences for socio-economic development. Women in developing countries lose more disability-adjusted life years from maternal health-related causes than to any other cause [10]. Thus, an understanding of the underlying factors contributing to maternal deaths is critical for targeted actions at both local and national levels [11, 12].

Generating accurate mortality data which is a critical component of public health information infrastructure can be achieved with the use of facility-based MPDSR in low and middle income countries [13]. Over 60% of low and middle income countries (LMICs) have a dysfunctional system of Civil Registration (CR), which is often regarded as the gold standard for producing reliable data on maternal deaths [14]. A study conducted in India revealed a significant underreporting of maternal deaths [15]. This is very crucial as each unrecorded maternal death is a missed opportunity to prevent similar deaths in the future [16]. Studies done on the impact of the MPDSR on maternal health outcomes suggest that maternal death reviews produce significant reductions in the incidence of obstetric complications and maternal mortality [17, 18, 19]. Other studies have highlighted the importance of maternal death reviews and surveillances in improving health providers’ knowledge, enhancing workforce capacity, encouraging professionalism, and scaling-up quality of care and resource mobilization [20, 21, 22, 23, 24].

Facility-based death review has been identified as one of the most effective methods to improve the performance of health systems needed for the sustained prevention of maternal mortality in Nigeria [6, 7]. In efforts to reducing the high maternal mortality rate in the country, Nigeria's Federal Ministry of Health (FMOH) in 2013 approved that all maternal health institutions in the country should periodically carry out maternal death reviews, surveillance and response using the technical guidance document as recommended by the World Health Organization [25]. This was updated in 2016 to include Perinatal Death Reviews, while the initiative was re-titled Maternal and Perinatal Death Surveillance and Response (MPDSR), to take account of the equally high rates of stillbirth, neonatal and perinatal deaths in the country [25]. Essentially, it was hoped that with regular reviews of maternal deaths and an analysis of the causes of deaths, that recommendations could be made that if addressed, would reduce the high rates of maternal mortality in the country [26]. This approach was required to address the current lack of substantive data on the circumstances under which maternal deaths occur in Nigeria, needed to design strategies, policies, and programs at a health systems level to reverse the trend. In our initial report using this approach in Lagos, Nigeria, we were able to identify the background causes of maternal deaths in three referral hospitals [7]. However, we were unable to show substantive policy response which prevented the documentation of the cycle of events necessary to demonstrate the effectiveness of this process.

The objective of this study was to report on the experiences in initiating and establishing the MPDSR in Central Hospital (CH), Benin City, and the policy response associated with the reporting of causes of maternal deaths in the hospital. CH is a secondary care referral hospital, which we had previously shown to have one of the highest maternal mortality ratios among eight referral hospitals in four geopolitical zones of the country [8]. We believe that this report will be useful in providing a framework for engaging policymakers to address the bottlenecks that lead to high rates of maternal mortality in the country.

## Methods

### Study area

The study was conducted at the Central Hospital (CH) in Benin City, Edo State, one of the 36 States in Nigeria. Nigeria operates a three-tier health care system with primary health care as the first tier. The second and third tiers are referral hospitals comprising secondary hospitals as the second tier and tertiary or teaching hospitals as the third tier. The Central Hospital Benin City is the main secondary referral hospital established more than 60 years ago in Benin City that has an estimated population of over 1.7million people. The hospital offers comprehensive antenatal, delivery and post-natal care as well as Comprehensive Emergency Obstetric Care (CEOC).

### Study design

This study is a part of a lager quasi-experimental research on improving the quality of emergency obstetric care for preventing maternal and perinatal mortality in referral hospitals in Nigeria. At the baseline of the larger project, a high maternal mortality ratio of 2992 per 100, 000 live births was observed at Central Hospital Benin [8]. Establishing a MPDSR was identified as one of the strategies to reduce maternal mortality in that hospital.

The current study was conducted in three Phases. In Phase I, we conducted strategic planning sessions with the senior management staff of the hospital, as well as policymakers at the State Ministry of Health that supervise the hospital. We broached the issue of the high rate of maternal mortality and conducted a SWOT (strength, weakness, opportunities, and threats) analysis to identify the bottlenecks that needed to be addressed in the hospital to resolve the problem and initiated a strategic operation plan to improve emergency obstetric care delivery and reduce maternal mortality ratio (MMR) by 50% in two years. A consensus was reached among all stakeholders (policymakers of the State ministry of health and senior management staff of the hospital) involved in the strategic planning process to work towards reducing the maternal mortality ratio in the hospital within two years. One of the activities identified as critical to achieve this outcome was the establishment and implementation of a policy on compulsory review of all maternal deaths that occur in the hospital. Such deaths would be reviewed for medical and contributory causes of mortality, with the idea to rectify the identified causes to avert future deaths in the hospital.

In the second phase of the study, we conducted a needs assessment of the knowledge of clinical staff about MPDSR, which showed poor knowledge and non-availability of the service in the hospital. Thereafter, we trained all staff of the maternity section of the hospital on the processes and methods of the MPDSR using the Federal Ministry of Health training protocol [25]. This was followed by the establishment of the MPDSR Committee of the hospital following the guidelines for establishing such committees approved by the Nigerian Federal Ministry of Health [25]. This Committee was made up of Medical Director of the hospital as the Chairman, the Head, Obstetrics and Gynaecology as Secretary, Heads of Departments of Nursing/Midwifery, Paediatrics, Pathology, Anesthesia, Hematology and Blood Bank, Labour/Maternity ward, Medical Records, and Pharmacy as members. An advocacy team that will notify the relevant stakeholders in the State Ministry of Health of the findings of the MPDSR committee was also constituted.

The third phase of the project was carried out over the next 20 months (October 2017 to May 2019). During this period, the MPDSR Committee of the hospital held bimonthly meetings and reviewed consecutive maternal deaths that occurred in the hospital. As part of the procedure, the case notes and associated information relating to each maternal death were retrieved by the Secretary of the Committee as soon as the deaths occurred. These were preserved privately until the reviews were conducted. The reviews were conducted confidentially. Only members of the committee were allowed into the room, while all information obtained in connection with the reviews were handled confidentially, and in a value-free manner. The names and contact details of the women who suffered maternal deaths and the names of the health providers that attended to the women were not revealed during the meetings. The reviews were conducted in a “no-blame manner” as recommended by the Federal Ministry of Health. It was designed only to explore the true medical, socio-economic and situational reasons for the death, so as to obtain information to prevent future deaths in the hospital [S1 File].

### Study population

All maternal deaths that occurred at the facility from October 2017 to May 2019 were included in this study.

### Research instrument

The nationally approved MPDSR tool (semi-structured, pretested and validated data collection questionnaire) was used for the notification of the deaths. The information required in the questionnaire was entered by the Secretary of the MPDSR Committee and a timely review of the deaths was conducted by the MPDSR committee.

### Data collection methods

Data was collected from the case files of the deceased women as soon as the death occurred and entered into the questionnaire attached in the MPDSR document by the Secretary of the MPDSR Committee. Data collected included pregnancy—related characteristics of the deceased women, time of admission to the hospital, gestational age at time of death, duration of hospital stay and medical and contributory causes of death.

**Fig 1:**
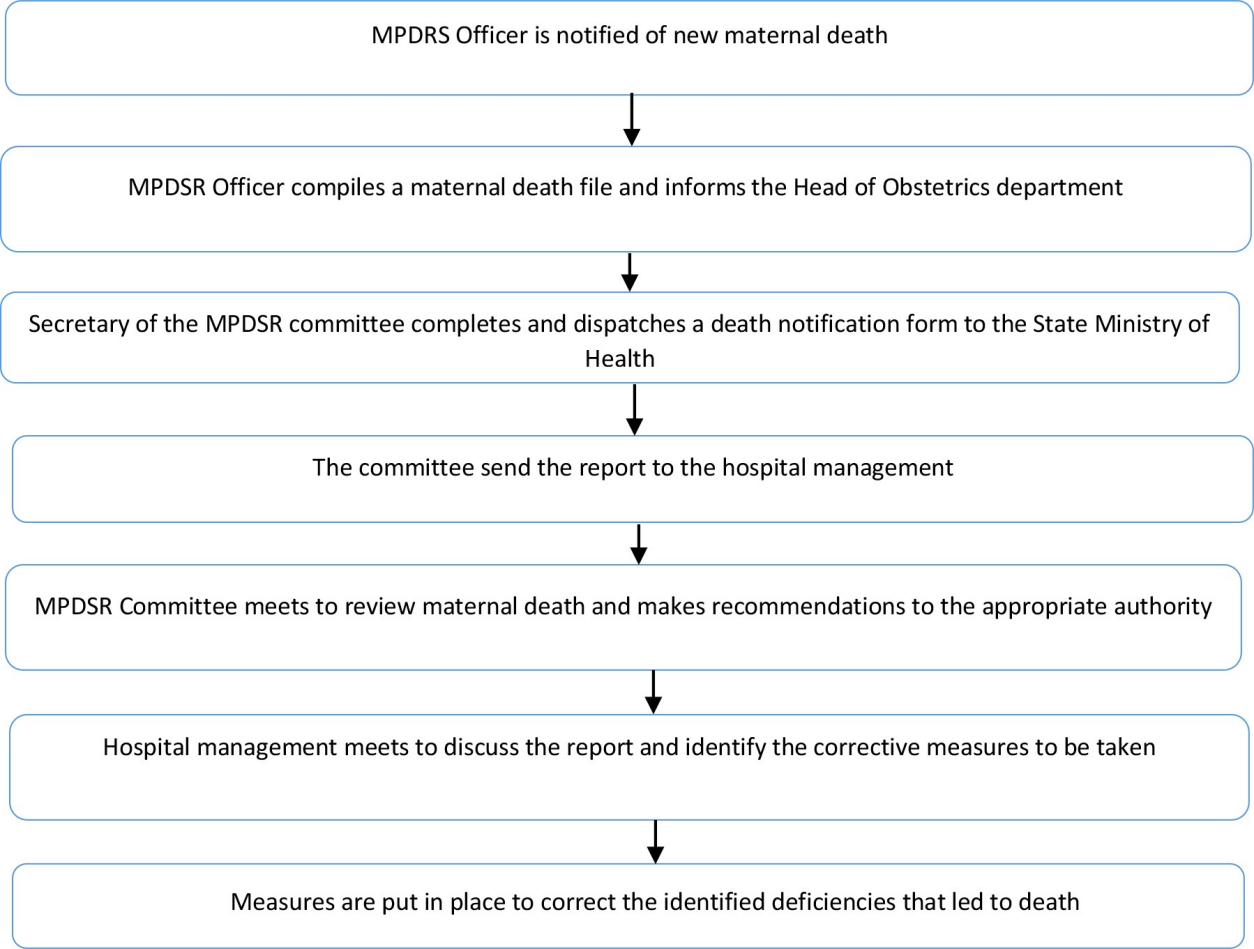
A flow chart showing the MPDSR process used in the study.

Following the reviews, the medical causes, as well as the contributory factors associated with the deaths were identified. The committee made recommendations to the head of the institution and to the State government for rectifying the identified medical and contributory causes of deaths. An advocacy committee consisting of two members of the research team, the Head of Department of Obstetrics and Gynecology, and a senior nursing officer was constituted to follow up with the various Departments of the Hospital and the State to ensure that the recommendations are put into appropriate policies and actions. A flow chart of the MPDSR process is presented in ‘‘Fig 1“.

Using a data extraction form designed for this study, the researchers collected data from the MPDSR committee immediately after each review meeting. We extracted data from the MPDSR questionnaire on the pregnancy-related characteristics of the deceased, the identified medical causes, contributory factors, and the recommendations. Data on policy responses were collected as documented by the committee. We also collected data on the number of deliveries during the period, the number of maternal deaths, and the proportion of women who died that received antenatal care in Central hospital (booked cases) versus those who did not receive antenatal care in the hospital (unbooked cases).

### Data analysis

The SPSS software 21.0 was used in analyzing the data. To ensure the validity of the data entered, double entry and random checks were carried out. Univariate analysis was done and presented using frequency tables. We calculated the maternal mortality ratio (MMR) as number of maternal deaths per 100,000 live births in the hospital.

To identify the nature of recommendations made for averting maternal mortality in the hospital we compiled the recommendations from the MPDSR committee and analyzed them qualitatively for form, theme, and content. The results were presented qualitatively.

We also presented reports of ways that the government and policymakers responded to the recommendations made, and the specific policy actions put in place to address the bottlenecks that were identified during the review.

### Ethical approval

Ethical approval for the study was obtained from the World Health Organization and the National Health Research Ethics Committee (NHREC) of Nigeria–number NHREC/01/01/2007–16/07/2014, renewed in 2015 with NHREC 01/01/20047-12/12/2015b.

The Chief Medical Director of the Hospital and the Head of the Department of Obstetrics and Gynaecology were informed of the purpose of the study, and consent was obtained from them to conduct the review and the study. No names or specific contact information were obtained from the study participants.

Ethical approval for the study was also obtained from the Edo State Ethical Review Board, as well as consent from the Edo State Ministry of Health. Additionally, all data were anonymized before access to the researchers and the MPDSR committees.

## Results

### Pregnancy-related characteristics of maternal deaths

From October 2017 to May 2019, a total of 18 maternal deaths were reviewed, while 4,557 deliveries were recorded in the hospital for the period. The maternal mortality ratio during the period was therefore 395 per 100,000 deliveries. As shown in Table 1, the ages of the women who died ranged from 18 to 42 (median = 31) years. The median parity was 2 (range, 0–11), and the median gestational age was 38.0 weeks (range, 8–42). The median length of hospital stay was 2 days (range, 0–24) days. A majority (77.8%) of the women died during the immediate postpartum period.

**Table 1:** Pregnancy-related characteristics of maternal deaths at the Central Hospital, Benin City (October 2017 –May 2019).

Variables	Frequency (n = 18)	Percentage (%)
**Age (in Years)**		
18–29	5	27.8
30–42	13	71.2
**Parity**		
Para 0–1	4	22.2
Para 2–4	8	44.4
Para > 5	6	33.3
**Gestational age (in Weeks)**		
24–26	2	11.1
27–36	-	-
Above 37	16	88.9
**Referral status**		
Not referred (women who were registered with the hospital)	7	38.8
Referred from other private health facilities	5	27.8
Referred from Traditional Birth Attendants (TBAs)	5	27.8
Referred from a religious home	1	5.6
**Gestational time of death**		
Antenatal	2	11.1
Intrapartum	2	11.1
Postpartum	14	77.8
**Duration of stay in hospital before death**		
Within 24 hours of admission	8	44.4
Between 1 & 2 days of admission	5	27.8
Between 3 & 7 days of admission	2	11.1
After one week of admission	3	16.7

Of the 18 deaths, 7 (seven) were not referred (booked cases); this means that they were registered with the hospital and had attended antenatal (ANC) clinic while 11 (61%) had been admitted as obstetric emergencies in the hospital (unbooked cases). The 11 unbooked cases had been referred from private clinics, traditional birth attendants (TBAs), and religious homes. The most common reasons for referrals included: 1) intensive care management; 2) further workup and management; 3) emergency high-risk treatment for all the 11 cases; and 4) blood transfusion in 6 cases. Of the 18 maternal deaths, 8 occurred during the first 24 hours of admission at the facility; 5 occurred between one and two days; 2 occurred between three and seven days; while the remaining 3 women died after one week of admission in the hospital.

### Obstetric causes of maternal deaths

The International Classification of Diseases–Maternal Mortality (ICD–MM) classification system was applied by the MPDSR committee in identifying the medical causes of death for all the cases reviewed. The medical cause of death was defined as the disease or condition that initiated the morbid chain of events that led to death [27].

The main medical causes identified in the 18 maternal deaths were obstetric hemorrhage (n = 10), pulmonary embolism (n = 2), ruptured uterus (n = 2), eclampsia (n = 1), anemic heart failure (n = 1) and post-partum sepsis (n = 2) (Table 2).

**Table 2:** Obstetric causes of maternal deaths at Central Hospital, Benin City.

Primary cause of death	Frequency (n = 18)	Percentage (%)
Post–partum Hemorrhage	10	55.5
Eclampsia	1	5.6
Anemic heart failure	1	5.6
Ruptured Uterus	2	11.1
Pulmonary embolism	2	11.1
Post–partum sepsis	2	11.1

### Contributory factors to maternal deaths

The MPDSR identified several background factors that contributed to maternal deaths. As shown in Table 3, these were broadly categorized into bottlenecks in the hospital, and factors associated with the women or their families. Interestingly both types of background factors were identified in all the 18 maternal deaths. As shown, the lack of blood in the hospital was the predominant hospital related contributory factor, followed by a delay in commencing treatment, the lack of essential drugs and non-functional intensive care unit.

**Table 3:** Contributory factors to Maternal Deaths in Central Hospital, Benin City from October 2017 to May 2019.

**Contributory facility-based factors**	**Frequency** **(n = 18)**	**Percentage** **(%)**
Delay in commencing treatment	5	27.8
Delay in deciding to refer patients	1	5.6
Non–functional Intensive Care Unit (ICU)	2	11.1
Non–availability of blood products when needed	6	33.3
Lack of skilled manpower	2	11.1
Lack of essential emergency drugs	2	11.1
**Contributory patient-based factors**	**Frequency** **(n = 18)**	**Percentage** **(%)**
Delay in reporting to health facility	5	27.8
Poor case management from referral facilities	8	44.4
Financial constraints	3	16.6
Patient refusal of treatment on admission	1	5.6
Poor compliance with treatment	1	5.6

By contrast, the major patient contributory factor was women first reporting in inappropriate delivery locations (e.g. the homes of TBAs) and then reporting late in the hospital with severe emergency obstetric complications. This occurred in eight cases, out of which blood was not immediately available for transfusion in six women. Other patient associated factors identified from the reviews included delay in the women arriving in hospital from their homes, lack of funds to pay for services, and non-compliance with treatment protocols.

### Recommendations to policymakers

Arising from the results of the reviews, the MPDSR committee made substantive recommendations to the management of the hospital to rectify various bottlenecks. The recommendations made are shown in Table 4. These included ensuring the increased availability of blood and blood products, the recruitment and re-training of staff, improvement of the intensive care unit, public health education of women and their relatives, and engagement with TBAs and referring hospitals to ensure early referral of women in difficult situations. The committee also recommended that the CH signs a cooperative agreement with the tertiary hospital in the city, the University of Benin Teaching Hospital to ensure the prompt secondary referral of cases that they cannot immediately manage. The recommendations were made through a formal letter signed by the Chairperson of the Committee to the Chief Medical Director of the hospital with copies to other senior medical officials, the Director of Health Services and the Commissioner for Health in the State.

**Table 4:** Recommendations on Preventing Maternal Deaths at the Central Hospital, Benin City and Policy Response.

S/N	Recommendations
1	Recruitment/deployment of more doctors, nurses and anesthesiologist to the Intensive Care Unit (ICU) at the facility.
2	Ensure the availability of blood products at all times.
3	Regular training and retraining of health workers on basic and comprehensive emergency obstetric care.
4	Revitalize the emergency Cesarean Section (CS) pack system at the facility.
5	Regular sensitization of members of the public through paid radio and television advertisements on the importance of antenatal care uptake, complying with medical advice and delivering at a health facility.
6	Ensure proper supervision of Traditional Birth Attendants (TBAs) and mission houses by the relevant monitoring agencies.
7	Proper use of case management protocols for all obstetric cases.
8	Develop a synergy between the facility and the tertiary health facility in the State, the University of Benin Teaching Hospital (UBTH), to ensure the speedy transfer of referred patients in emergency situations.

### Policy response

We also monitored the policy response to the recommendations made by the MPDSR committee. Some of the policies and actions that were taken in the hospital in response to the recommendations of the committee included: 1) more attention was given to issues relating to maternal deaths in the hospital; 2) the institution of a monthly maternal health education meetings in the hospitals involving pregnant women, their husbands/family members, medical and nursing staff, management staff and policymakers; 3) the establishment of a blood donation drive in partnership with the State Blood Transfusion Service; 4) the refurbishing of the Intensive Care Unit in the hospital; and 5) the general mobilization and sensitization of staff to handle pregnant women with great sensitivity, promptness and care.

## Discussion

The objective of this paper is to report the results of the MPDSR conducted at the CH Benin City, Nigeria using a nationally approved protocol, and to characterize the related policy actions. In particular, we intended to demonstrate how a systematic process of reviews and reporting of the medical and social causes can lead to specific recommendations that can be acted on by policymakers to address the identified causes of mortality.

In 2015, the Nigerian Federal Ministry of Health recommended that all 36 States of the country should adopt the MPDSR as a way to identify the specific causes of maternal mortality, and then to rectify the bottlenecks that lead to death [25]. By addressing such factors, it was expected that this would lead to a significant decline in maternal mortality rates in the country. While the use of maternal death reviews and surveillance has been found to be effective in many developed countries for averting maternal deaths, [23, 28, 29] this approach has not been systematically used in Nigeria. To the best of our knowledge, this is the first report of the full use of the MPDSR in Nigeria that went through the complete cycle of identification of maternal deaths, review of the medical and social causes of deaths, the recommendations made to policymakers/managers, and the actual follow-up to ensure that specific remediating actions are taken.

It was of interest to note that once the initial results of the MPDSR were made available to policymakers, appropriate action was taken and new measures were put in place to provide additional resources including staff deployments, staff training, the provision of maternal health education to women and their families, and improved availability of blood in the hospital. Eighteen women died in the hospital during the 20 month period covered by the MPDSR, with a maternal mortality ratio (MMR) of 395/100,000 deliveries. However, this is a drastic reduction when compared to the MMR of 2,992/100,000 deliveries which we documented in the hospital prior to the commencement of the review [8]. Although it is not the intention of this paper to report the effects of MPDSR on MMR, nonetheless, it is noteworthy that the hospital witnessed a substantial decline in mortality rate during the period of the review. Future follow-up assessments will be needed to determine the true effects of MPDSR on maternal mortality in the country.

It is likely that the success of this initial MPDSR process at the Central Hospital can be attributed to three mechanisms. First, the initial strategic plan conducted for stakeholders and policymakers of the hospitals helped to build consensus and commitments for reducing the high rate of maternal mortality in the hospital. This ensured that all activities carried out with respect to the MPDSR were closely monitored and received the support of hospital managers and stakeholders. Secondly, the “no blame” and confidential approach utilized for the MPDSR ensured the full cooperation of all staff involved in the process. Additionally, the concurrent training provided to all staff during the review provided further understanding and enabled the re-awakening of staff about the steps needed to reduce maternal mortality in the hospital [30].

Thirdly, the follow-up and advocacy activities that were carried out after the submission of the MPDSR recommendations to managers and policymakers ensured that specific actions were taken to rectify the identified bottlenecks. We believe that without such follow-up activism and what amounted to evidence-based advocacy using the results of the MPDSR it would have been difficult to achieve the reported positive outcomes. It is evident therefore that MPDSR will only be successful when stakeholders are mobilized intensely to understand the process, enabling them to collaborate in implementing the process and in taking appropriate remediating actions when the results are provided [19, 20].

One of the major difficulties of implementing an obstetric audit program in low- income countries is the poor quality of data collected [15]. There are many sources of errors based on retrospective data recording and of interpretation, errors from disorganization in patients’ files, and errors of tallying and reporting. The use of prospective data collection and the commitment of staff in documenting cases of maternal death through the MPDSR seeks to address these problems. This is a major strength of this study. A limitation of this study was the initial fear of blame on the part of the health care providers. However, we were able to overcome this by ensuring that all committee meetings were conducted in a no blame, no name atmosphere. This paper presents a description of the process of establishing a MPDSR committee, the identified causes and contributory factors, recommendations and policy response. We recommend further studies on the evaluation of the MPDSR process.

We conclude that MPDSR has helped to classify the causes of maternal deaths, to identify avoidable factors, and to make recommendations for changes in professional practice at the Central Hospital, Benin City. Through the careful engagement of managers, policymakers and relevant stakeholders throughout the entire review cycle, we have demonstrated that it is possible to immediately rectify associated bottlenecks and therefore reduce high rates of maternal mortality.

## Supporting information

S1 File Central Hospital MPDSR Summary. (DOCX)
