# Supplementary material for: Maternal death review and surveillance: The case of Central Hospital, Benin City, Nigeria
Source: PLoS One. 2019 Dec 19;14(12):e0226075. doi: 10.1371/journal.pone.0226075 (PMC6922332; doi:10.1371/journal.pone.0226075)
Supplement: S1 File — (DOCX) [file pone.0226075.s001.docx]

**CENTRAL HOSPITAL, BENIN CITY**

**SUMMARY OF MPDSR COMMITTEE RECORDS**

**NOVEMBER 2017 – MAY 2019**

| **S/N** | **AGE** | **PARITY** | **GESTATIONAL AGE (WKS)** | **NO OF DAYS ON ADMISSION** | **REFERRAL STATUS** | **OBSTETRIC CAUSE OF DEATH** | **CONTRIBUTORY CAUSE OF DEATH (FACILITY BASED)** | **CONTRIBUTORY CAUSE OF DEATH (PATIENT BASED)** | **RECOMMENDATION (FACILITY)** | **RECOMMENDATION (GOVERNMENT)** | **RESPONSE** |
| --- | --- | --- | --- | --- | --- | --- | --- | --- | --- | --- | --- |
| 1. | 30 | 1 | 37 | 6 | Referred from a private clinic | Severe PPH With Anaemia | Refusal of the blood bank to give blood to the patient without payment | Financial constraints | Blood products should always be available for emergencies | Organize extensive health education campaigns on social media and other platforms on the importance of regular blood donation from members of the public | The M.D of the facility has set up a panel of enquiry to investigate the issue with the blood bank because there is an ongoing scheme of free blood donation in partnership with the State Blood Transfusion Service from where blood could initially be given out to patients who are in dire need. |
| 2. | 28 | 1 | 26 | <1 | Referred from a private clinic | Severe PPH Occasioned By Ruptured Ectopic Pregnancy | Non-functional Intensive care Unit at the facility | Poor case management of patient before referral | A sub-committee should be set up to look into the non-usage of the ICU at the facility | Recruit adequate manpower to the ICU at the facility | The ICU at the facility has been refurbished and a new ventilator and monitor have been bought. |
| 3. | 42 | 5 | 38 | 2 | Referred from a TBA | Severe PPH due to uterine ATONY | Non-availability of blood products | Poor case management of patient before referral | A massive blood drive should be reorganized by the facility | Efforts should be made to sensitize more women on the uptake of ANC and discourage their patronage of TBAs. | Copies of Case Mgt. Protocols for managing PPH, Eclampsia and the third stage of Labour has been developed and formally presented to the facility by WHARC. |
| 4. | 30 | 1 | 40 | 2 | Registered with CH | Thrombo-embolism | Delay in commencing treatment | Financial constraints | Proper use of case management protocol, especially in cases of emergencies | Purchase equipment and refurbish the Labour ward theatre | The resuscitier and anesthetic machine in the Labour ward theatre have been replaced, the operating light and an air conditioning unit have been replaced also. |
| 5. | 40 | 4 | 38 | <1 | Referred from a TBA | Ruptured uterus due to prolonged labour | Non-functional Intensive Care Unit at the facility | Poor case management of patient before referral | Incentives should be given to the TBAs (as in the case of Ondo State), to encourage them to promptly refer pregnant women to specialized health care facilities. | Use of women’s groups and NGOS to advocate for prompt use of the hospitals and discourage TBAs | Paid advertisement has been put up on radio and television to inform more people about the monthly comprehensive couples’ talk at the facility to sensitize more women on the benefits of ANC. |
| 6. | 35 | 5 | 38 | 2 | Registered with CH | Severe PPH | Non-availability of blood products | Delay in reporting to health facility | Blood products should always be available for emergencies | Facility should organize comprehensive health education campaigns on social media and other platforms on the importance of regular blood donation from members of the public | A monthly maternal health education meeting of pregnant women and their spouses has been initiated at the facility. |
| 7. | 38 | 4 | 41 | 2 | Referred from a religious home | Severe PPH | Non-availability of blood products | Poor case management of patient before referral | Make blood and blood products available at all times | Organize extensive health education campaigns on social media and other platforms on the importance of regular blood donation from members of the public | The facility has established a blood donation drive in partnership with the State Blood Transfusion Service to ensure the availability of blood products at all times |
| 8. | 32 | 3 | 37 | 14 | Referred from a TBA | Ischemic cerebrovascular disease secondary to eclampsia | Lack of essential drugs and other consumables | Poor case management before referral | Pharmacy should make emergency drugs available in the Labour ward at all times | Revitalize the emergency CS pack system in the facility | The emergency CS pack system from the pharmacy to the Labour ward is currently on-going at the facility. |
| 9. | 29 | 5 | 38 | <1 | Referred from a private clinic | Hemorrhagic shock due to severe PPH | Non availability of blood products | Poor case management of patient before referral | Laboratory department should always ensure that blood products are available | The State Ministry of Health should create a means of engaging TBAs and other smaller private clinics to sensitize them on the need for early referral of women in difficult situations in order to avoid mortalities | The M.D of the facility directed his secretary to write a letter to the First Lady of the State to inform her on the rate of morbidities and avoidable mortalities from unregistered health facilities and untrained personnel in order for her to device the means to regulate their activities. |
| 10. | 32 | 2 | 41 | <1 | Registered with CH | Thrombo-embolism | Delay in commencing treatment by the health personnel on duty (there was a 4 hour delay before commencing surgery) | Refusal of treatment as patient initially declined caesarean section | Enforce proper use of case management protocol | The facility should always engage in the public health education of women and their relatives in order to prevent avoidable mortalities | There has been a reorientation workshop organized for the healthcare workers at CH to sensitize them on the need to respond quickly to emergency situations |
| 11. | 30 | 6 | 40 | <1 | Registered with CH | Hypovolemic shock due to severe PPH | Delay in deciding to refer patient to the Teaching Hospital | Delay in reporting to health facility | Laboratory department should always ensure that blood products are available | Modalities should be put in place to secure a synergy between the facility and the tertiary health facility in the state (UBTH) to ensure speedy transfer of patients in emergency situations | A Memorandum of understanding has been signed with the management of UBTH to facilitate immediate response and care to patients referred from CH |
| 12 | 40 | 1 | 38 | <1 | Referred from a TBA | Hypovolemic shock occasioned by Severe PPH | Lack of skilled manpower to promptly attend to emergencies | Delay in reporting to the facility on time as patient was brought in from a rural area after she had delivered at a TBA | Address the issue of brain drain in the facility | The State Ministry of Health should educate the people on the dangers of patronizing TBAs. | Ongoing sensitization programs in the State to educate women on the need for proper self-care and healthy behaviours at all times especially during pregnancy |
| 13. | 35 | 6 | 38 | 8 | Registered with CH | Hemorrhagic shock due to severe PPH | Delay in commencing treatment | Financial constraint | There should be proper use of case management protocol | The facility should organize extensive health education campaigns on social media and other platforms on the importance of regular blood donation from members of the public | The HOD, Blood Bank has given the directive that all maternal cases needing blood should be attended speedily to even without initial deposit from the patient |
| 14. | 28 | 4 | 26 | <1 | Referred from a TBA | Toxic shock occasioned by Puerperal sepsis | Lack of essential drugs and other consumables | Poor compliance with treatment | Revitalize the emergency CS pack system in the facility | The State Ministry of Health should educate the people on the dangers of patronizing TBAs. | Ongoing sensitization programs in the State to educate women on the need for proper self-care and healthy behaviours at all times especially during pregnancy |
| 15. | 30 | 4 | 38 | <1 | Registered with CH | Anemic Heart failure | Delay in commencing treatment | Delay in reporting to the health facility | There should be proper use of case management protocol | The facility should always engage in the public health education of women and their relatives in order to prevent avoidable mortalities | The facility is currently organizing a general mobilization and sensitization of staff to handle pregnant women with great sensitivity, promptness and care |
| 16. | 29 | 3 | 41 | 2 | Registered with CH | Post-partum hemorrhage occasioned by Abruptio placenta | Unavailability of patient’s blood type in the blood bank | Delay in reporting to the health facility | The management of the Blood Bank should ensure that all blood types/groups are available at all times | Request Has Been Made To The State Ministry Of Health to purchase an Apheresis machine for blood fractioning and also provide appropriate storage equipment | The advocacy team has paid an advocacy visit to the Commissioner of Health in the State to inform him of this request**.** |
| 17. | 28 | 3 | 38 | 4 | Referred from a private clinic | Cardiopulmonary failure secondary to Puerperal sepsis | Lack of skilled manpower to respond quickly to emergencies | Poor case management of patient before referral | There is need for the recruitment of more staff and the regular sensitization of patients on the need for regular antenatal checkup. | Address the issue of brain drain in the facility especially with the new law of retirement after 35 years of service. | The State Government is now committed to improving maternal health in the facility and has redeployed more healthcare providers to the maternity unit of the facility |
| 18. | 32 | 5 | 40 | 10 | Referred from a private clinic | Ruptured uterus due to prolonged labour | Delay in commencing treatment | Poor management of patient before referral | There should be proper use of case management protocol | The massive and continuous sensitization of all women in the society on the importance of early uptake of antenatal care. | The facility is currently organizing a general mobilization and sensitization of staff to handle pregnant women with great sensitivity, promptness and care |
